
JOURNAL INFORMATION
==============================
NLM Title Abbreviation: Behav Neurol
Journal ID: Behav Neurol
NLM Journal ID: 8914585
Journal ID: BN

Behavioural Neurology

ISSN: 0953-4180
EISSN: 1875-8584
Publisher: Wiley

ARTICLE INFORMATION
==============================
PMCID: PMC5507117
DOI: 10.1155/2002/806405
Article ID: 806405
Article version: 1
Subjects: Book Review

Limbic Seizures in Children, by G. Avanzini, A. Beaumanoir and L. Mira, Eds

Ferrie C. D.
Yorkshire Regional Paediatric Neurology Servive United Leeds Teaching Hospitals NHS Trust Clarendon Wing Room 81 Leeds General Infirmary Leeds LS2 9NS UK
Print publication date: 2002
Electronic publication date: 2002 Nov 1
Volume: 13
Issue: 3-4
First page: 149
Last page: 149
Received 2002 Sep 28; Accepted 2002 Sep 28
Copyright: Copyright © 2002 Hindawi Publishing Corporation.
Copyright year: 2002
License: This is an open access article distributed under the Creative Commons Attribution License, which permits unrestricted use, distribution, and reproduction in any medium, provided the original work is properly cited.
License URL: https://creativecommons.org/licenses/by/3.0/

==============================
Behavioural Neurology 13 (2001/2002) 149
IOS Press

### Book review

**Limbic seizures in children**, Edited by G. Avanzini, A. Beaumanoir and L. Mira

This volume is the latest in a series of texts on different aspects of paediatric epilepsy produced by the Mariani Foundation in Italy and published by John Libbey & Co. Previous volumes have been well received within the epileptology community and in seeking to address the question of limbic disease in children the editors have sought to address an important area of childhood epileptology and one which is both difficult and controversial.

The book is based on a series of presentations given at a Colloquium on the same subject. It is multi-authored. A large majority of the authors have direct or indirect links with the Marseilles School of Epileptology. This can certainly be viewed as a strength given the prodigious output of high quality work from this school. However, one might have hoped for a greater involvement from other well-respected centres.

The text begins with a detailed and very interesting historical account by Beaumanoir and Roger. There then follows a series of chapters addressing fundamental aspects of the anatomy and physiology of the limbic system. This in itself is a major undertaking given the complexity of the structure and the controversies that exist as to its definition. As a clinician I found these chapters extremely interesting and at times illuminating, but also heavy going.

There then follows a series of chapters which address in considerable detail various aspects of the symptomatology of limbic epilepsies with particular emphasis on those occurring in children. Further chapters cover the role of and the insights to be gained from neurophysiological, structural imaging, and functional imaging in limbic epilepsies, aspects of the etiology of limbic epilepsies, the medical and surgical treatment of limbic epilepsies, and finally a synopsis which seeks to bring the various contributions together.

As is the case for all the preceding texts in this series each chapter is in effect an essay in its own right. The chapters are not intended to tell a coherent story but rather to offer insights, sometimes contradictory, into different aspects of the subject matter. Each author uses a different style and indeed different methodology. Some of the chapters consist of detailed reviews of previous work, others present new findings, some combine both approaches and others are mainly concerned with personal viewpoints, albeit viewpoints arrived at after a wealth of experience. This is both a strength and a weakness. The book does not yield ready answers. It is not a reference book, nor is it a clinical guide. Rather it is a scholarly treatise whose principal audience is likely to be those with a specialist interest in epileptology. Researchers from many disciplines, including psychologists, physiologists, neuro-pharmacologists, and clinical epileptologists will find this a valuable addition to their library. Those who are seeking straightforward answers to clinical questions are, however, likely to be disappointed.

*C.D. Ferrie*
Yorkshire Regional Paediatric Neurology Servive
United Leeds Teaching Hospitals NHS Trust
Clarendon Wing
Room 81, Leeds General Infirmary
Leeds, LS2 9NS, UK

ISSN 0953-4180/01/02/$8.00 © 2001/2002 – IOS Press. All rights reserved
